# Rapid Onset of Clinical Response to Eculizumab in a Single-Center Cohort of Older Patients with Refractory Generalized Myasthenia Gravis

**DOI:** 10.3390/neurosci7040085

**Published:** 2026-07-24

**Authors:** Giulia D’Alvano, Salvatore Del Giudice, Francesca D’Anna, Vincenzo Todisco, Alessandro Tessitore, Alvino Bisecco

**Affiliations:** 1 Department of Advanced Medical and Surgical Sciences, University of Campania “Luigi Vanvitelli”, 80138 Naples, Italy; giulia.dalvano@unicampania.it (G.D.); delgiudicemd@gmail.com (S.D.G.); francesc.danna1994@gmail.com (F.D.); alessandro.tessitore@unicampania.it (A.T.); 2 First Division of Neurology and Neurophysiopathology, AOU University of Campania “Luigi Vanvitelli”, 80138 Naples, Italy; vincenzo.todisco@policliniconapoli.it

**Keywords:** myasthenia gravis, eculizumab, real-world evidence, elderly

## Abstract

Background: Current treatments for myasthenia gravis (MG), while improving outcomes, are often associated with adverse effects, and a proportion of patients remain refractory to standard therapies. Eculizumab, a humanized monoclonal antibody targeting complement protein C5, is approved for refractory anti-acetylcholine receptor antibody-positive generalized MG (AChR-Ab+ gMG). Aim of the present study is to explore the rapidity of onset of eculizumab efficacy in a single-center cohort of MG patients. Methods: This retrospective, observational, single-center study evaluated the real-world effectiveness and rapidity of response to eculizumab in patients with AChR-Ab+ gMG. Patients received eculizumab according to the approved regimen and were assessed at baseline (T0), 5 weeks (T1), and 3 months (T2) using the Myasthenia Gravis Activities of Daily Living (MG-ADL) score, Quantitative Myasthenia Gravis (QMG) score, and Myasthenia Gravis Foundation of America (MGFA) classification. Results: Eight patients were eligible for the study with a mean age of 73.1 years. A rapid clinical improvement was observed after treatment initiation. Mean MG-ADL scores decreased from 8.38 at T0 to 2.13 at T1 (*p* < 0.001; Cohen’s d = 2.075), while QMG scores declined from 13.75 to 5.63 (*p* = 0.001; Cohen’s d = 1.824). Improvements were maintained at T2. Mean percentage reductions from T0 to T1 were 73.5% for MG-ADL and 60.6% for QMG. Eculizumab was well tolerated. Conclusions: These real-world findings confirm a rapid and clinically meaningful response to eculizumab in elderly patients with refractory gMG. These findings highlight its potential role in achieving early symptom control and reducing disease burden in this clinically vulnerable population.

## 1. Introduction

Myasthenia gravis (MG) is an autoimmune disorder of the neuromuscular junction (NMJ) affecting the postsynaptic membrane [1]. The main clinical manifestation of MG is fatigable muscle weakness, which can potentially involve any skeletal muscle. The primary therapeutic objective is to achieve a state of minimal manifestations status (MMS), with no symptoms or functional limitations, or better, thereby enhancing the patient’s quality of life [2]. The management of MG has historically relied on acetylcholinesterase inhibitors for symptomatic relief, alongside corticosteroids and broad-spectrum immunosuppressants (IS). While these conventional immunosuppressive strategies achieve satisfactory symptom control for a substantial proportion of patients, their utility is often limited by a delayed onset of action, sometimes requiring weeks to reach full therapeutic effect, and a significant burden of long-term systemic side effects [2,3]. This therapeutic landscape presents a considerable challenge, as approximately 10–15% of individuals with generalized MG (gMG) are classified as refractory [4].The pathophysiology in the most common form of MG involves autoantibodies against the acetylcholine receptor (AChR-Ab), which mediate damage at the NMJ through several mechanisms [5]. Among these, the activation of the classical complement pathway, culminating in the formation of the lytic membrane attack complex (MAC) on the postsynaptic membrane, plays a crucial role [6,7,8].

The critical role of the complement system in AChR-Ab+ MG pathogenesis has spurred the development of targeted therapies. Eculizumab, a humanized monoclonal antibody, binds to complement protein C5, preventing the cleavage of C5 into its active fragments, C5a and C5b, thereby inhibiting the terminal complement cascade and the ensuing MAC-mediated NMJ damage [9]. The landmark REGAIN trial provided robust evidence for the efficacy and safety of eculizumab in patients with refractory AChR-Ab+ gMG, demonstrating significant clinical improvements over placebo, with most of the treatment effect occurring by week 12 [10]. Eculizumab was first approved by the United States Food and Drug Administration (FDA) for the treatment of refractory AChR-Ab+ MG in 2017 [11], followed by approval from the European Medicines Agency (EMA) in the same year [12].

Following the approval of eculizumab in Italy by Italian Medicines Agency (AIFA) in 2022 [13], we conducted a single-center, observational retrospective study. The primary objective of the study was to evaluate the rapidity of onset and clinical magnitude of the improvement following eculizumab therapy in refractory patients, alongside a comprehensive assessment of its overall efficacy and tolerability, in a real-world setting. Furthermore, this investigation aimed to characterize the therapeutic response, thereby informing potential future research into the role of eculizumab in managing patients needing rapid onset of action, such as those with impending myasthenic crisis, acute disease exacerbations, or clinical situations in which prolonged exposure to high-dose corticosteroids is contraindicated, poorly tolerated, or associated with an unacceptable risk of adverse effects.

## 2. Materials and Methods

### 2.1. Study Design and Setting

This was a retrospective, observational, single-center study conducted at the First Division of Neurology and Neurophysiopathology of the University of Campania “Luigi Vanvitelli” in Naples, Italy. Data from patients treated between October 2023 and July 2024 were retrospectively collected.

### 2.2. Study Population

Patients with a diagnosis of refractory gMG who were positive for anti-AChR antibodies and underwent treatment with eculizumab were included.

The inclusion criteria were:

- A diagnosis of gMG refractory to conventional immunosuppressive treatment
- Positivity for anti-AChR antibodies, confirmed by radio-immunoassay (RIA) and/or cell-based assay (CBA).
- Vaccination against Neisseria Meningitidis (serogroups A, C, Y, W135 and B) at least 14 days before the start of therapy or, when not immediately available, prophylactic antibiotic treatment until the end of the vaccination cycle, in accordance with current recommendations for complement inhibitor therapy.
- MG-ADL score at baseline ≥ 6, in accordance with the AIFA reimbursement criteria for eculizumab [13].
- Duration of treatment with eculizumab ≥ 3 months.

Refractory gMG was defined as failure to achieve adequate symptom control despite treatment with corticosteroids and at least two other IS agents, or one IS agent along with chronic IVIg/PLEX, or intolerance to these therapies, consistent with AIFA criteria for eculizumab reimbursement [13].

Exclusion criteria were:

- Ocular or generalized non-refractory MG.
- MG positive for anti-MuSK or anti-LRP4 antibodies, or seronegative.

### 2.3. Treatment Protocol

All patients received eculizumab according to the standard approved posology for gMG: an induction phase consisting of 900 mg intravenously (IV) weekly for the first 4 weeks, followed by a first maintenance dose of 1200 mg IV at week 5. Subsequent maintenance doses of 1200 mg IV were administered every 2 weeks (±2 days).

### 2.4. Data Collection and Outcomes

Baseline demographic and clinical characteristics were recorded.

Clinical data were retrospectively collected from patient medical records at three timepoints:

- Baseline (T0): Immediately prior to the first eculizumab infusion.
- Post-induction (T1): 5 weeks after the first eculizumab infusion (after completion of the 900 mg weekly induction and the first 1200 mg dose).
- Follow-up (T2): after 3 months from baseline.

The primary efficacy outcomes were the change from baseline in the Myasthenia Gravis Activities of Daily Living (MG-ADL) score (range 0–24, higher scores indicate greater impairment) [14] and the Quantitative Myasthenia Gravis (QMG) score (range 0–39, higher scores indicate greater impairment) [15]. Changes in the Myasthenia Gravis Foundation of America (MGFA) clinical classification [16] and adverse events (AEs) occurring during the observation period were also recorded, as well as data on concomitant medications.

### 2.5. Statistical Analysis

Descriptive statistics, including mean and standard deviation (SD) for continuous variables and frequencies and percentages for categorical variables, were used to summarize patient characteristics and outcome measures.

To assess longitudinal changes in the MG-ADL and QMG scores, paired-samples *t*-tests were conducted. Specifically, comparisons were made between T0 and T1, and between T1 and T2. The mean percentage change in MG-ADL and QMG scores from T0 to T1 was also calculated to quantify the initial treatment response.

Effect sizes for the significant paired *t*-test comparisons (T0 vs. T1) were calculated using Cohen’s d, based on the standard deviation of the difference scores, with effects interpreted as small (0.2), medium (0.5), or large (0.8).

A *p*-value of <0.05 was considered statistically significant for all analyses.

All statistical analyses were performed using Jamovi version 2.6 [17].

## 3. Results

### 3.1. Baseline Characteristics

A total of eight patients were included in the study. Baseline demographic and clinical characteristics are summarized in Table 1. The cohort was evenly distributed by sex, with four (50.0%) males and four (50.0%) females. The mean age at study enrollment was 73.1 (±6.7 years). The mean age at disease onset was 66.8 (±13.2 years), and the mean age at diagnosis was 66.9 (±14.2 years). Regarding the type of MG onset, two patients (25.0%) presented with an ocular onset, while six patients (75.0%) had a generalized onset. All patients (100.0%) were positive for anti-AChR antibodies. A history of thymoma was reported in two patients (25.0%), all of whom had undergone thymectomy.

Regarding concomitant MG treatments, all patients (100.0%) received pyridostigmine, 6 (75.0%) received prednisone, and 1 (12.5%) received an IS in addition; 4 (50.0%) patients had a history of treatment with IS, suspended for adverse events. Mean dose of prednisone at baseline was 17.5 mg/day. Two patients (25.0%) had a history of myasthenic crisis in the year preceding treatment with eculizumab, both treated with IVIg. The most frequent comorbidities included hypertension (5 patients, 62.5%), dyslipidemia (3 patients, 37.5%), and hyperuricemia (3 patients, 37.5%).

At baseline, 6 patients (75.0%) were classified as MGFA class IIIa, 1 patient (12.5%) as class IIIb, and 1 patient (12.5%) as class IVb. The mean baseline MG-ADL score was 8.38 ± 2.67, and the mean baseline QMG score was 13.75 ± 6.76.

### 3.2. Efficacy Outcomes

The mean follow-up duration for the cohort was 14.7 (±3.5) weeks.

#### 3.2.1. MG-ADL and QMG Scores

Treatment with eculizumab resulted in rapid and statistically significant improvements in both MG-ADL and QMG scores from baseline.

**Figure 1:**
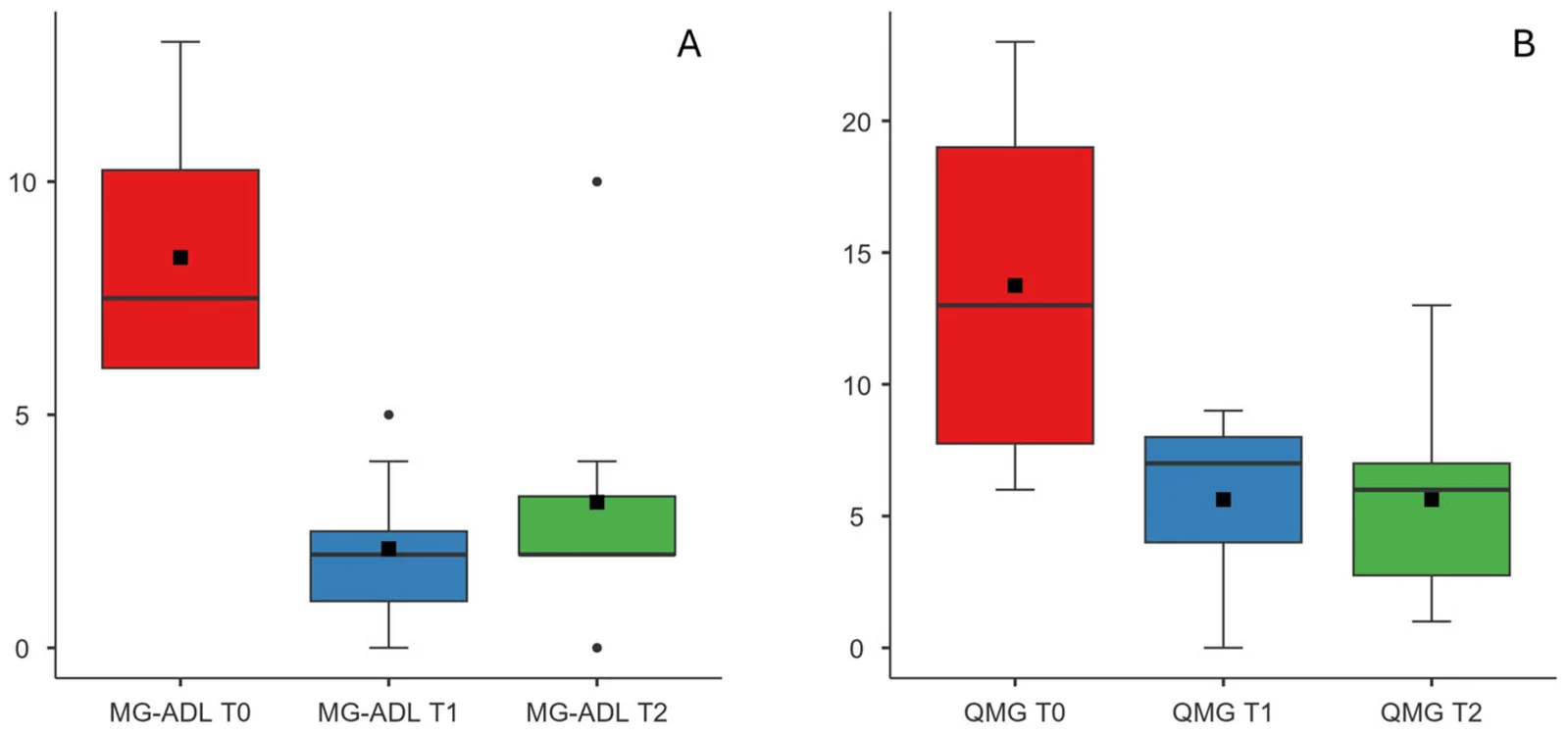
Changes in clinical scores over time with eculizumab treatment (*n* = 8). Box plots illustrating the distribution of MG-ADL (**A**) and QMG (**B**) scores at baseline (T0), 5 weeks post-induction (T1), and 3 months from baseline (T2). Individual data points beyond the whiskers are shown as outliers. The black square within each box indicates the mean value.

A significant reduction in MG-ADL scores was observed at T1 (Figure 1A): the mean MG-ADL score decreased from a baseline (T0) of 8.38 (±2.67) to 2.13 (±1.64) at T1 (*p* < 0.001, Cohen’s d = 2.075). In particular, all patients (100%) achieved a reduction ≥ 2 points in MG-ADL score, corresponding to the established Minimal Clinically Important Difference (MCID) for MG-ADL [18] (Table 2). At 3 months (T2), the mean MG-ADL score was 3.13 (±3.00). There was no statistically significant difference in mean MG-ADL scores between T1 and T2 (*p* = 0.17).

Similarly, a significant reduction in QMG scores was evident at T1 (Figure 1B). The mean QMG score decreased from a baseline (T0) of 13.75 (±6.76) to 5.63 (±3.38) at T1 (*p* = 0.001, Cohen’s d = 1.824). Specifically, in 7/8 patients (87.5%) a reduction ≥ 3 points in QMG score—corresponding to the MCID [19]—was observed (Table 2). At 3 months (T2), the mean QMG score was 5.63 (±3.81), showing no significant change compared to T1 (*p* = 1.00).

**Figure 2:**
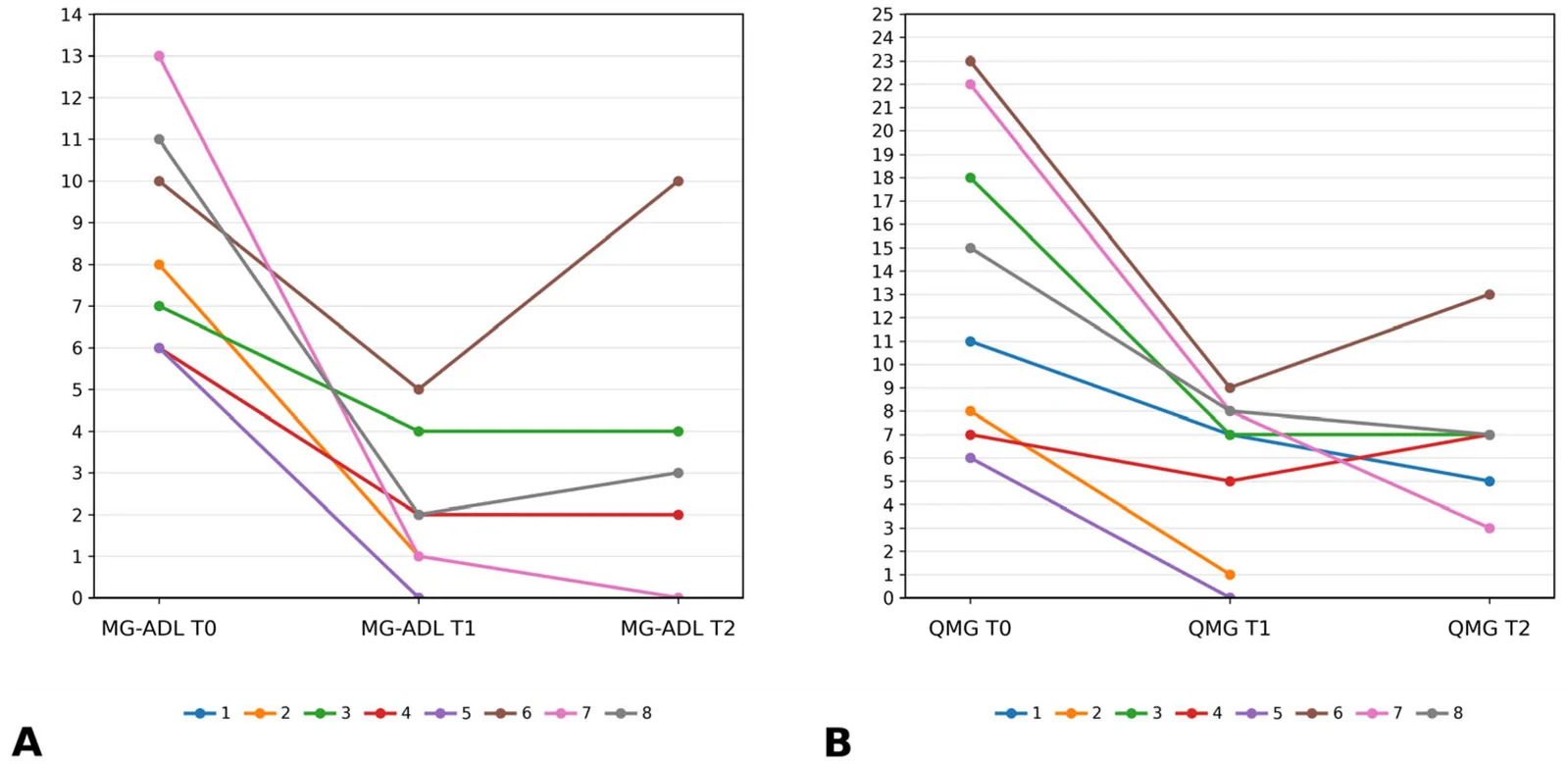
Individual changes in clinical scores during eculizumab treatment. Line plots showing individual patient trajectories for MG-ADL (**A**) and QMG (**B**) scores at baseline (T0), 5 weeks post-induction (T1), and 3 months from baseline (T2). Each line represents a single patient, illustrating the variability and overall trend of clinical response over time.

Individual changes in MG-ADL and QMG scores are shown in Figure 2.

**Figure 3:**
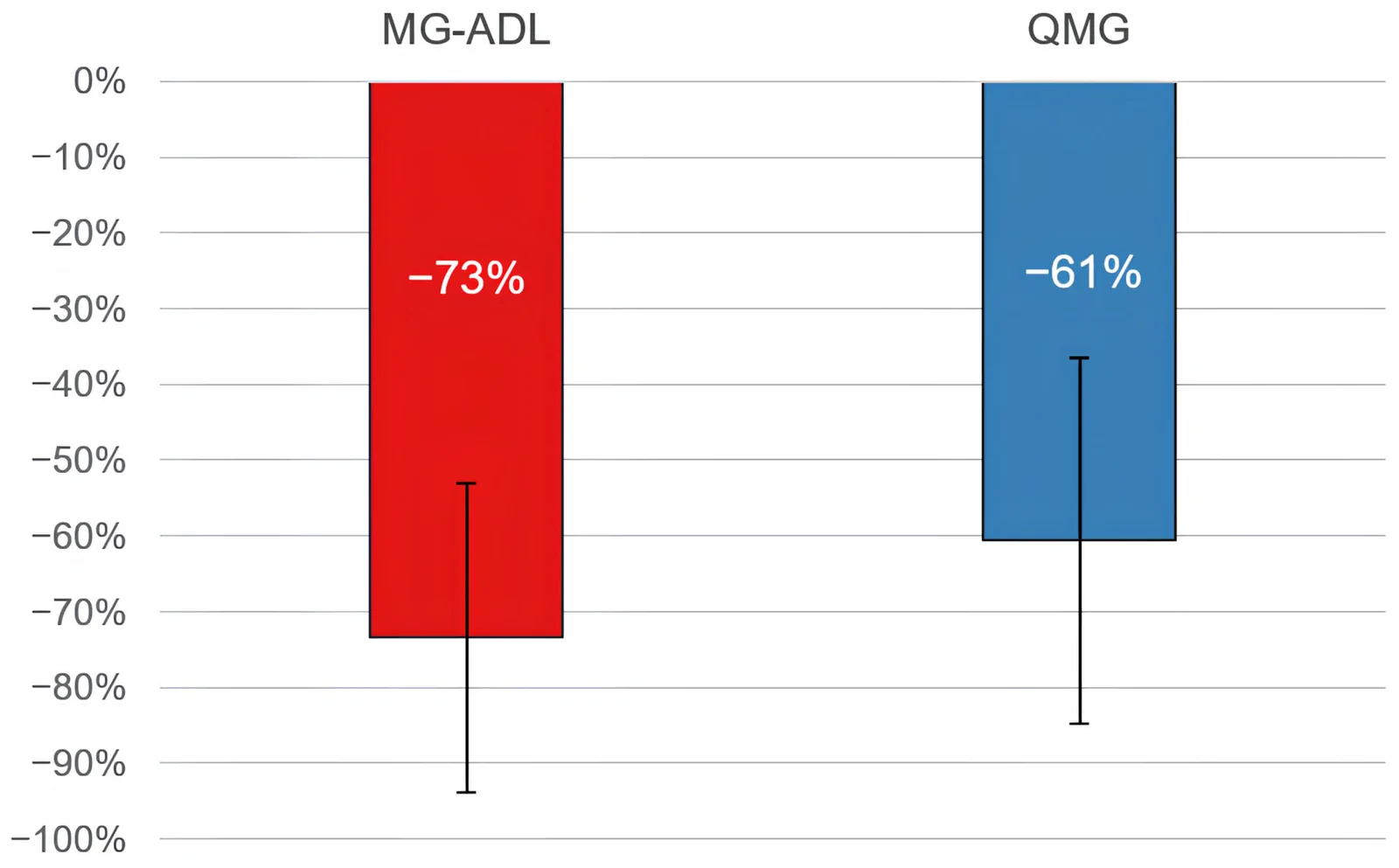
Mean percentage change in MG-ADL and QMG scores from baseline (T0, *n* = 8) to 5 weeks post-induction (T1, *n* = 8).

The rapid clinical benefit was further highlighted by the mean percentage reduction in scores from T0 to T1. The MG-ADL score decreased by a mean of −73.5% (±20.4%), and the QMG score decreased by a mean of −60.6% (±24.2%) (Figure 3).

#### 3.2.2. MGFA Classification

Improvement in MGFA classification was observed in all eight patients at T1 (5 weeks). Specifically, four patients improved from class IIIa to class IIa; two patients improved from class IIIa to class I; one patient improved from class IIIb to class IIa; and one patient improved from class IVb to class IIa. At T2, these improvements were largely maintained or consolidated, with two patients worsening by one subclass (from class IIA to class IIB, and from class IIA to class IIIA), while six patients remained stable.

### 3.3. Concomitant Medication, Safety and Tolerability

All 8 patients included in the study received vaccination against *Neisseria meningitidis* (serogroups A, C, Y, W135 and B) prior to the initiation of eculizumab. No cases of meningococcal infection were reported during the observation period. Similarly, no other serious opportunistic infections were observed during the study period.

Eculizumab was generally well-tolerated. Three patients (37.5%) reported no AEs during the follow-up period. Five patients (62.5%) experienced at least one AE. The reported AEs were headache (2 patients, 25.0%), community-acquired pneumonia (2 patients, 25.0%), and COVID-19 infection (1 patient, 12.5%). All reported AEs were managed according to standard clinical practice.

No patient discontinued eculizumab due to AEs. Furthermore, no patient experienced a clinical exacerbation or required rescue therapy (such as IVIg or PLEX) during the eculizumab treatment period evaluated in this study.

No relevant changes in concomitant immunosuppressive treatment were recorded during follow-up, except for a 12.5 mg/day reduction in prednisone dosage in one patient.

## 4. Discussion

This single-center, real-world study showed that eculizumab provides rapid and substantial clinical improvement for patients with refractory anti-AChR+ gMG.

While the efficacy of eculizumab in refractory AChR-positive gMG is now well established [10,20], the timing of clinical response remains particularly relevant in routine practice. Although early clinical responses to eculizumab have been reported in both clinical trials and real-world cohorts, few studies have specifically focused on the magnitude of improvement achieved within the first weeks of treatment in refractory AChR-positive gMG. Patients with severe disease burden often require prolonged exposure to corticosteroids, repeated rescue therapies, or hospitalization while awaiting the effects of conventional immunosuppressive agents [3]. In this context, the marked reduction in MG-ADL and QMG scores observed as early as 5 weeks post-treatment initiation suggests that complement inhibition provides clinically meaningful improvement within a relatively short timeframe. The ability to quickly reduce symptoms can lead to more immediate gains in daily function and quality of life, potentially lessening the psychological impact of the disease and reducing reliance on long-term immunosuppression.

Beyond statistical significance, the magnitude of the observed effect was considerable, as reflected by large effect sizes observed for both MG-ADL and QMG outcomes. This finding suggests that the clinical improvement was not only rapid but also substantial, reinforcing the relevance of the observed changes for everyday functioning.

Our results are consistent with findings from the pivotal REGAIN trial, which also reported early clinical benefits of eculizumab, generally within 4 to 12 weeks [10]. Following its approval, several real-world studies have confirmed the efficacy of eculizumab in MG. Jin et al. demonstrated efficacy and safety of eculizumab in a cohort of patients with thymoma-associated MG, reporting a significant improvement in the MG-ADL score at week 12 [21]. Similarly, Pane et al. observed a reduction in the MG-ADL score after only one week of treatment, and in the QMG score after 5 weeks [22].

The rapid clinical response observed in our cohort is further supported by emerging evidence from patients with severe disease manifestations. Several case reports and small case series have described the successful use of eculizumab as rescue therapy in refractory or impending myasthenic crisis, often documenting rapid neurological improvement and early liberation from mechanical ventilation [22,23,24,25,26]. In particular, Vinciguerra et al. reported complete clinical recovery and ventilator weaning within days of treatment initiation [23], while Strano et al. described substantial respiratory and motor recovery within weeks despite prolonged failure of conventional rescue therapies [24]. More recently, Erra et al. reported favorable outcomes in a series of patients treated with eculizumab as second- or third-line rescue therapy for refractory or impending crisis, demonstrating its efficacy after 1 or 2 weeks [25]. Although evaluated in a different clinical context, the rapid improvement observed in our patients appears consistent with this growing body of evidence.

Unlike conventional immunosuppressive therapies, which require weeks or months to modulate adaptive immune responses [1], complement inhibition acts directly on one of the final effector pathways responsible for neuromuscular junction damage in AChR-positive MG [7]. This mechanism may explain why clinical benefits can emerge shortly after treatment initiation.

From a clinical perspective, the rapidity of response may represent an important factor when selecting among available targeted therapies for refractory gMG. In our cohort, clinically meaningful improvements in both patient-reported daily functioning and objective muscle strength were already evident at the first assessment, only 5 weeks after treatment initiation. Notably, all patients achieved the MCID for MG-ADL, while 87.5% reached the MCID for QMG, suggesting a consistent early benefit across complementary outcome measures. This may be particularly relevant in patients with a high disease burden, in whom rapid symptom control could potentially reduce the need for rescue therapies and limit prolonged exposure to high-dose corticosteroids. However, direct comparisons with other targeted therapies cannot be inferred from our observational data and would require appropriately designed head-to-head studies.

Of note, our cohort had a higher mean age than those reported in previous real-world studies [21,22,25]. Although often underrepresented in clinical trials [10,20], elderly patients constitute an increasing proportion of MG patients and represent a particularly challenging therapeutic group. Age-related factors, including multimorbidity, polypharmacy, frailty, and increased vulnerability to treatment-related adverse events, frequently influence therapeutic decisions in this population [27,28]. In particular, the use of conventional immunosuppressive therapies may be limited by their delayed onset of action and the risk of cumulative toxicity, especially infections and metabolic complications associated with prolonged corticosteroid exposure. Moreover, a French multicenter retrospective study highlighted a substantial iatrogenic burden associated with immunosuppressive treatment in elderly MG patients, including severe and fatal infectious complications [29]. In this setting, treatment decisions are often influenced by the balance between disease control and the tolerability of long-term immunosuppression.

For the abovementioned reasons, the availability of targeted therapies with a more selective mechanism of action represents a relevant therapeutic advance for older patients with refractory disease. Emerging real-world evidence on targeted therapies, including complement inhibitors and FcRn antagonists, suggests that these treatments may retain clinical effectiveness in older patients with gMG, with an acceptable safety profile, although dedicated studies in elderly populations remain limited [30,31].

The advanced age of our cohort is therefore of particular interest, as our findings suggest a rapid and clinically meaningful response to complement inhibition, even in older patients. The significant improvement observed within the first weeks of treatment may be especially relevant in elderly patients, in whom prolonged periods of uncontrolled disease may result in functional decline, increased dependence, and an increased risk of complications. Moreover, the favorable tolerability observed in our cohort, despite concomitant immunosuppressive treatments and the presence of age-related comorbidities, provides additional real-world evidence supporting the feasibility of eculizumab treatment in this setting. Nevertheless, these observations should be interpreted cautiously given the limited sample size and the observational nature of the study, and larger prospective studies are needed to better define the effectiveness, safety, and optimal positioning of eculizumab in elderly patients with refractory gMG.

In our cohort, eculizumab was generally well tolerated, with an adverse event profile consistent with previous studies. No meningococcal infections or other serious infectious complications were observed during the study period, despite concomitant immunosuppressive therapy. The reported adverse events were manageable, and importantly, no patients discontinued eculizumab because of adverse events or required rescue therapy for MG exacerbation during follow-up.

The primary strength of this study is its contribution of real-world data on the early impact of eculizumab in a clinically challenging refractory MG population. However, the study has limitations inherent to its retrospective, single-center design and small sample size, which affect the generalizability of the findings. These methodological issues impose caution in the interpretation of the results and underscore the need for further prospective, controlled, multicenter studies conducted on larger sample sizes to confirm and generalize our observations. Furthermore, open questions remain, particularly regarding the optimal duration of treatment, cost-effectiveness, and long-term tolerability. The rapid efficacy of eculizumab also suggests a plausible rationale for exploring its utility in acute MG exacerbations, a direction that merits dedicated prospective, controlled, multicenter trials with larger patient numbers.

## 5. Conclusions

These real-world findings support the rapid onset and sustained effectiveness of eculizumab in patients with AChR-Ab+ refractory gMG, including older patients, highlighting its potential role in achieving early symptom control and reducing disease burden in this particularly vulnerable population. Our findings align with the efficacy and safety data from the REGAIN study and support further investigation into the potential use of this drug for managing acute exacerbations of the disease.

## Figures and Tables

**Table 1:** Baseline demographic and clinical characteristics.

Baseline Characteristics	*n* = 8
**Gender, *n* (%)**	
Female	4 (50%)
Male	4 (50%)
**AChR-Ab+, *n* (%)**	8 (100%)
**Mean age at enrollment, years (±SD)**	73.1 (±6.7)
**Mean age at diagnosis, years (±SD)**	66.9 (±14.2)
**Average duration of illness, years (±SD)**	6.3 (±7.5)
**Type of onset, *n* (%)**	
Ocular	2 (25%)
Generalized	6 (75%)
**MGFA class, *n* (%)**	
IIIa	6 (75%)
IIIb	1 (12.5%)
IVb	1 (12.5%)
**Thymoma, *n* (%)**	2 (25%)
Thymectomy	(2/2) (100%)
**Concomitant treatments, *n* (%)**	
Pyridostigmine	8 (100%)
Prednisone	6 (75%)
(mean dose)	17.5 mg/day
MMF	1 (12.5%)
**Previous myasthenic crisis, *n* (%)**	2 (25%)
Use of IVIg	(2/2) (100%)
**Most frequent comorbidities, *n* (%)**	
Hypertension	5 (63%)
Dyslipidemia	3 (38%)
Hyperuricemia	3 (38%)
Cataracts	2 (25%)
Glaucoma	2 (25%)
Hypovitaminosis D	2 (25%)

**Table 2:** Outcome measures at T1.

Outcome at T1	*n* (%)
MG-ADL improvement ≥ 2 pt	8 (100)
QMG improvement ≥ 3 pt	7 (87.5)
MGFA improvement ≥ 1 class	8 (100)
MGFA class I at T1	2 (25)
Rescue therapy needed	0 (0)

## Data Availability

The data presented in this study are available on request from the corresponding author because they contain clinical data from human participants and are subject to privacy, ethical, and institutional restrictions. Public deposition of the complete dataset could compromise participant confidentiality and is not permitted under the conditions of the Ethics Committee approval and applicable data protection regulations. An anonymized data table containing the data underlying the analyses was uploaded during the manuscript submission process for editorial evaluation. Additional anonymized data supporting the findings of this study are available from the corresponding author upon reasonable request, subject to applicable ethical and institutional regulations.

## Abbreviations

The following abbreviations are used in this manuscript:

AE	Adverse Event
AChR	Acetylcholine Receptor
AChR-Ab	Anti-Acetylcholine Receptor Antibody
AZA	Azathioprine
CBA	Cell-Based Assay
gMG	Generalized Myasthenia Gravis
IS	Immunosuppressant(s)
IV	Intravenously
IVIg	Intravenous Immunoglobulin
MAC	Membrane Attack Complex
MCID	Minimal Clinically Important Difference
MG	Myasthenia Gravis
MG-ADL	Myasthenia Gravis Activities of Daily Living
MGFA	Myasthenia Gravis Foundation of America
MMF	Mycophenolate Mofetil
MMS	Minimal Manifestations Status
NMJ	Neuromuscular Junction
PLEX	Plasma Exchange
QMG	Quantitative Myasthenia Gravis
RIA	Radio-Immunoassay
SD	Standard Deviation
